# Semi-Supervised Generative Adversarial Nets with Multiple Generators for SAR Image Recognition

**DOI:** 10.3390/s18082706

**Published:** 2018-08-17

**Authors:** Fei Gao, Fei Ma, Jun Wang, Jinping Sun, Erfu Yang, Huiyu Zhou

**Affiliations:** 1 School of Electronic and Information Engineering, Beihang University, Beijing 100191, China; mafeimf@buaa.edu.cn (F.M.); wangj203@buaa.edu.cn (J.W.); sunjinping@buaa.edu.cn (J.S.); 2 Strathclyde Space Institute, Department of Design, Manufacture and Engineering Management, University of Strathclyde, Glasgow G1 1XJ, UK; erfu.yang@strath.ac.uk; 3 Department of Informatics, University of Leicester, Leicester, LE1 7RH, UK; hz143@leicester.ac.uk

**Keywords:** Generative Adversarial Networks (GANs), semi-supervised recognition, Synthetic Aperture Radar (SAR), deep learning

## Abstract

As an important model of deep learning, semi-supervised learning models are based on Generative Adversarial Nets (GANs) and have achieved a competitive performance on standard optical images. However, the training of GANs becomes unstable when they are applied to SAR images, which reduces the feature extraction capability of the discriminator in GANs. This paper presents a new semi-supervised GANs with Multiple generators and a classifier (MCGAN). This model improves the stability of training for SAR images by employing multiple generators. A multi-classifier is introduced to the new GANs to utilize the labeled images during the training of the GANs, which shares the low level layers with the discriminator. Then, the layers of the trained discriminator and the classifier construct the recognition network for SAR images after having been finely tuned using a small number of the labeled images. Experiments on the Moving and Stationary Target Acquisition and Recognition (MSTAR) databases show that the proposed recognition network achieves a better and more stable recognition performance than several traditional semi-supervised methods as well as other GANs-based semi-supervised methods.

## 1. Introduction

Compared with visible light based systems, infrared, or other remote sensing techniques, SAR can achieve all-day and all-weather imaging for various geographical terrains, and therefore becomes one of the main means of extracting target information. Especially, the latest technology advance in SAR imaging sensors, such as Terra SAR-X and COSMO-SkyMed, has improved the imaging resolution greatly, providing more detailed features for target recognition [1,2]. Traditional recognition methods for SAR images includes the Support Vector Machine (SVM) [3], Fisher Linear Discriminant Analysis (FDA) [4] et al. With the introduction of deep learning [5,6], Deep Convolution Neural Networks (DCNN) have made important breakthroughs in many aspects of image processing [7,8], such as SAR image recognition. For example, Wagner et al. [9] proposed a recognition model using Convolution Neural Networks (CNN) (as the feature extractor) and SVM (as the classifier), achieving a 98% recognition accuracy on the MSTAR dataset. Wagner et al. [10] further improved the accuracy to 99% by adding Morphological Component Analysis (MCA) to the input layer of CNN as a preprocessing step. In addition, three types of data augmentation operations: Translation, Speckle Noising, and Pose Synthesis, were introduced into the training of DCNN networks [11], which further improves their recognition accuracy. However, most of these established classifiers require a large amount of labeled images for learning/training. Time consumption, in manually labeling images, limits their applications in practice.

To handle this problem, some unsupervised deep learning models have been proposed, such as the Restricted Boltzmann Machine (RBM) [12], the Sparse Auto-Encoder (SAE) [13], and the Deep Belief Network (DBN) [14]. These deep models attempt to capture the probability distributions over the given image space. However, their recognition accuracy for MSTAR is not satisfactory [15].

GANs is a new unsupervised learning model, first proposed by Goodfellow et al. in 2014 [16]. This model is composed of a generator (G) and a discriminator (D). The discriminator is a binary classifier and its objective is to determine whether the input images are real or generated by the generator. The generator attempts to generate fake images to deceive the discriminator. Two networks are trained synchronously until the discriminator cannot distinguish between real and fake images. The main applications of GANs includes the generation of visually realistic images [16], semantic image inpainting [17], and image super-resolution [18].

The adversarial game between the discriminator and the generator enable the discriminator to have a better feature extraction capability than RBM et al. Hence, more and more researchers have been attempting to apply GANs for semi-supervised recognition. The most common method was proposed by Salimans et al. [19]. In this model, the discriminator is modified to a K+1 class multi-classifier, where real images are assumed to have K classes and the faked images are regarded as having the (K+1)_th_ class. During the training, the labeled, unlabeled, and faked images are sent into the discriminator together. The objective of the discriminator is to assign the correct labels to the labeled real images and to classify the fake images as the (K+1)_th_ class. Similarly, Springenberg et al. [20] changed the discriminator to a multi-classifier. They redesigned the loss function from the perspective of the probability distribution of the multi-classifier output. The above method shave achieved promising results in the semi-supervised classification on MNIST (Modified National Institute of Standards and Technology database), CIFAR-10 (Canadian Institute For Advanced Research), SVHN (The Street View House Numbers), and other optical datasets. 

However, the standard GANs has no obvious advantages in SAR image classification against the standard DCNN methods. First, the highest current spatial resolution (the size of the smallest possible feature that can be detected) of SAR images is about 0.1 m, far lower than that of the current optical images. Lower resolution leads to subtler differences between different classes of SAR targets. These distinctions are further affected by the strong speckles from the coherence between radar echo signals, which greatly increases the instability of the GANs training. For example, Reference [21] reported experiments on the basic training DCGAN (Deep Convolutional Generative Adversarial Networks) [22] using SAR images, and found that the image quality from the generator frequently degraded, and even shattered into noise. The collapsing problem of GANs training reduces its feature learning capability, leading to low recognition accuracy. 

For now, most of the improved GANs solve the collapsing problem by modifying the loss functions of the generator and the discriminator, such as Wasserstein GAN (WGAN) [23] and Wasserstein GAN, with a Gradient Penalty (WGAN-GP) [24]. However, the discriminators for these methods are binary classifiers, which means the discriminators cannot learn category information during the training. 

**Figure 1:**
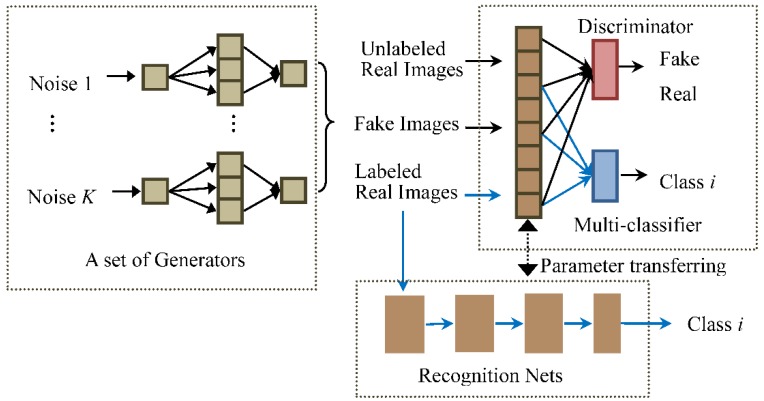
Architecture of our semi-supervised MCGAN and MCGAN-based recognition networks. The novel MCGAN contains three groups, a set of generators, a discriminator, and a multi-classifier, which are interactively trained. The multi-classifier network shares the feature extraction layers with the discriminator. Then, the low level layers from the discriminator and the multi-classifier constitute a recognition network for identifying SAR targets, which is further turned using the labeled images.

We here propose a novel GANs architecture with multiple generators to strengthen the capability of feature extraction of the discriminator for SAR images. Moreover, in order to let the discriminator learn class information from the labeled images, a multi-classifier is introduced in the GANs, which share feature extraction layers with the discriminator. We call the GANs, with Multiple generators and one multi-Classifier, the MCGAN. As shown in Figure 1, the training of MCGAN becomes an adversarial game for three parties: the generators aim to generate realistic SAR images; the classifier intends to attach the right labels to the labeled real images; and the discriminator determines whether or not the input is real.

In the application of semi-supervised recognition, the classifier and feature extraction layers of the discriminator together form the SAR target recognition network. The same set of the labeled images are used to fine-tune the network. The new network has better and more stable recognition performance than the standard supervised CNN, some improved GANs-based semi-supervised recognition models (DCGAN [22], WGAN-GP [24], DRAGAN [25]), and traditional semi-supervised models.

The rest of this paper is organized as follows: Section 2 introduces the framework of the original GANs and the improved GAN models. In Section 3, we describe the structure and learning process of our semi-supervised recognition network in detail. In Section 4, we conduct the classification experiments on the MSTAR datasets, and compare its results with some semi-supervised methods. A brief discussion on the stability of MCGAN during the training, from the viewpoint of mathematics, is presented in Section 5. Finally, the paper is concluded in Section 6.

## 2. Related Work

### 2.1. Generative Adversarial Networks

The original GANs mainly consist of a generator and a discriminator. The input of the generator is random noise z, with a distribution $P_{z}\left(z\right)$ (such as a Gaussian distribution), and it outputs faked images G(z) with the same size as that of the real images. The discriminator takes the real images x and the faked images as the input, where the real and the faked images are assumed to be subject to the distribution $P_{data}\left(x\right)$ and $P_{g}\left(x\right)$. If the input is real, the expected outputs of the discriminator D(⋅) are close to one; for the input G(z), the discriminator is expected to output a low probability (close to zero). The loss function of the training process is as follows:

(1) $$\underset{G}{\min}\;\underset{D}{\max}\;V\left(G,D\right)\;=E_{x\in P_{data}\left(x\right)}\left[\log D\left(x\right)\right]+E_{z\in P_{z}\left(z\right)}\left[\log\left(1-D\left(G\left(z\right)\right)\right)\right]$$

where $E_{x\in P_{data}\left(x\right)}\left[\log D\left(x\right)\right]$ represents the sum of logD(x) with various x obeying the distribution $P_{data}\left(x\right)$. On the right hand side, the first term of the right hand side of Equation (1) ensures that the discriminator can make proper judgment for the real training images (the output D(x) is close to one); the goal of the second term of the right hand side is to make the generated images G(z) as realistic as possible, so that the discriminator cannot distinguish them from the real images.

During the training, the first k steps aim to update the parameters of the discriminator using Stochastic Gradient Descent (SGD) with the condition that the parameters of the generator are fixed. More specifically, m noise items $\left\{z^{\left(i\right)},\ldots ,z^{\left(m\right)}\right\}$ enter the generator and become m fake images $\left\{G\left(z^{\left(i\right)}\right),\ldots ,G\left(z^{\left(m\right)}\right)\right\}$. Then, the faked and real images $\left\{x^{\left(i\right)},\ldots ,x^{\left(m\right)}\right\}$ are inputted into the discriminator, and the parameters of the discriminator are modified by maximizing the following loss function V(D):

(2) $$\underset{D}{\max}\;V\left(D\right)=\frac{1}{m}\sum_{i=1}^{m}\left[\log D\left(x^{\left(i\right)}\right)+\log\left(1-D\left(G\left(z^{\left(i\right)}\right)\right)\right)\right]$$

The (K+1)_th_ step trains the generator. Similarly, the m random noise $\left\{z^{\left(i\right)},\ldots ,z^{\left(m\right)}\right\}$ of the distribution $P_{z}\left(z\right)$ enters the generator to produce faked images $G\left(z^{\left(i\right)}\right)$. Then, the generator’s parameters are updated by minimizing the following loss function V(G):

(3) $$\underset{G}{\min}\;V\left(G\right)=\frac{1}{m}\sum_{i=1}^{m}\log\left(1-D\left(G\left(z^{\left(i\right)}\right)\right)\right)$$

Goodfellow et al. have proved that the GANs can obtain a global optimal solution after enough training, i.e., $P_{data}\left(x\right)=P_{g}\left(x\right)$. At this point, the discriminator and the generator cannot continue to improve in order to achieve the Nash equilibrium [16]. The discriminator’s output is $D\left(x\right)=D\left(G\left(z^{\left(i\right)}\right)\right)=\frac{1}{2}$.

### 2.2. Improved GANs Models

The training process of GANs often encounters the non-convergence problem, which is because the SGD can only ensure GANs achieves the Nash equilibrium when the loss functions are convex. The generator may collapse to a parameter setting where its outputs become very similar to each other. The discriminator believes the outputs of the generator are realistic and the gradient may push it to further learn these images. As a result, the GANs may never converge to an ideal Nash equilibrium.

Researchers have proposed a variety of new architectures to solve the instability problem in the training process of GANs. For example, Mirza et al. introduced CGAN (Conditional Generative Adversarial Nets) [26], where the class labels were added to the training dataset to train the generator. Then, Laplacian Pyramid of Generative Adversarial Network (LAPGAN) was introduced based on CGAN by combining GANs and Laplacian pyramids [27]. Each layer of the pyramid contains an independent generator and they share the same discriminator. The deeper the layers, the higher the resolution of the generated images are. The generator at the top layer generates the final faked images. 

**Figure 2:**
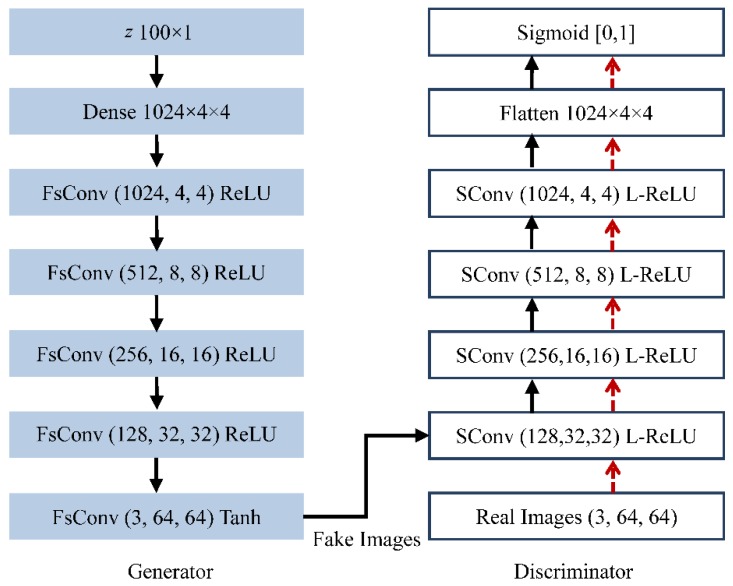
Architecture of DCGAN (Deep Convolutional Generative Adversarial Networks) for Faces database in Reference [22], where FsConv represents fractionally-strided convolutions; SConv is the acronym for strided convolutions; (1024, 4, 4) represents 1024 filters and the size of the convoluted image is 4 × 4; ReLU, Tanh, and L-ReLU are three types of activation functions, i.e., ReLU, Tanh, and Leaky-ReLU.

Radford et al. [22] proposed a new GANs model for generating images based on CNN (DCGAN). DCGAN achieves stable training for three optical databases (Large-scale Scene Understanding (LSUN), Imagenet-1k, and the latest Faces database) and can generate high quality faked images. Specifically, the architecture of DCGAN for the used Faces database is shown in Figure 2, where the size of each image is 64 × 64. The generator employs a fractionally-strided convolution layer, instead of an up-sampling layer, and removes the fully connected hidden layers. In the discriminator, the pooling layers are replaced by strided convolutions. The ReLU activation function is applied in both sub-networks, except in their output layers. Mao et al. proposed to replace it with an L2 Loss Function to improve the learning stability of GANs [28]. This modification enables the model to learn multi-class images, e.g., handwritten Chinese characters.

Recently, more GANs models were proposed to improve the stability of training by modifying the discriminator’s and generator’s objective functions. Generally, these methods all adopt the fractionally-strided convolutions and strided convolutions proposed in DCAGN to construct their GANs. For example, according to the theory of Reference [29] and Reference [23], training the generator in the original GANs is equal to minimizing the Jensen-Shannon divergence (JSD) between the distributions of the generated data and the real data. In practice, the overlapping part between the two distributions $P_{data}$ and $P_{g}$ is easily zero or negligible. As a result, the JSD will approach a constant (i.e., log2); the gradient of the loss function becomes zero and the generator stops learning from the real data. Therefore, Reference [23] proposed the Wasserstein GAN (WGAN), which introduced the Earth-Mover (EM) to replace the JSD to avoid the loss functions becoming constants. Then, WGAN-GP model further improved the convergence of WGAN by using the gradient penalty for enforcing the Lipschitz constraint instead of weight clipping [24].

Kodali et al. [25] analyzed the training of GANs from an entirely new angle and pointed out that the undesirable local equilibria were the reason for the collapse. The local equilibria usually have sharp gradients of the discriminator function around some real data points. They proposed a novel adaptive penalty scheme named DRAGAN (Deep Regret Analytic Generative Adversarial Networks) to avoid these local equilibria.

Another group of models intend to employ more generators to address the collapsing problem. For example, Reference [30] employed multiple generators in the GANs, which shared weights with each other. A multi-classifier is added in the GANs to recognize which generator the fake image comes from. This model could easily cover a variety of the real data distributions and effectively improve the stability of the training.

## 3. Materials and Methods

In this paper, we propose the MCGAN model and apply it with semi-supervised recognition for SAR images. In the first step, MCGAN is introduced to learn SAR images comprehensively. The low level layers of the trained discriminator transfers to a feature extractor of the recognition network, followed by a neural network classifier. Then, the same labeled SAR images are used to fine-tune the recognition network whilst passing the label information to the classifier.

### 3.1. The MCGAN for SAR Images

In general, the training of the GANs’ generator aims to find a distribution $P_{g}$ and make it have the real data distribution $P_{data}$ [23]. In traditional GANs training, we can always first obtain the optimal discriminator ($D^{*}$) [23]. Then, the generator is trained by minimizing the loss function, which can be represented as

(4) $$L\left(D^{*},g\right)=2JSD\left(P_{data}||P_{g}\right)-2\log2$$

where $JSD\left(P_{data}||P_{g}\right)$ represents the JSD of two distributions $P_{data}$ and $P_{g}$. It means that training the generator is equal to minimizing the JSD between the distributions of the generated data and the real data. The training process is only valid when there are overlaps between the two distributions. If the overlapping part is 0, the JSD will appear to be a constant, and the gradient of the loss function will become zero. The generator will stop learning. Therefore, stable training of the GANs networks is to ensure that the two distributions $P_{data}$ and $P_{g}$ have non-negligible intersections. However, It has been demonstrated in Reference [29] that for the standard GANs, the measure of their intersections equals 0 and its impossible for $P_{data}$ and $P_{g}$ to have non-negligible intersections.

In other words, if the discriminator performs better than the generator, the discriminator will give a probability of 1 for almost all the real samples, a probability of 0 for almost all the generated samples, and those parts that are difficult to classify will be ignored because their measure is 0. At this point, the loss function or the JSD of the two distributions is nearly a constant, and the generator’s gradient disappears. In practice, it is hard to control the learning speed of the discriminator. 

We propose to use *K* generators $G_{1:K}$ in the standard GANs. Assuming that the data distribution from the $k_{th}$ generator $G_{k}$ is $P_{G}{}_{k}$; the mixture distribution from the group of generators is $P_{\mathrm{model}}=\sum_{i=1}^{K}P_{G}{}_{i}$. To ensure that each $P_{G}{}_{k}$ is independent of each other, their weights are updated independently. When the discriminator is optimized ($D^{*}$) during the training, the optimal generator will be [30]

(5) $$G^{*}=\underset{G}{\min\arg}\left(2JSD\left(P_{data}||P_{\mathrm{model}}\right)\right)=\underset{G}{\min\arg}\left(\sum_{i=1}^{K}2JSD\left(P_{data}||P_{Gi}\right)\right)$$

Equation (5) indicates that the optimization of the generator is equivalent to finding the minimum JSD of real data distribution and mixture distribution $P_{\mathrm{model}}$. Compared with the GANs with one generator, the discriminator needs to simultaneously compete with multiple generators, thus reducing the convergence rate of the discriminator to render the optimal solution. As long as the discriminator does not reach the optimal performance, the loss function will not become a constant, so that the collapsing of the generators will not happen. Furthermore, multiple generators are trained individually and their respective data distributions are independent. Even if the discriminator has reached the optimality for the k_th_ generator $G_{k}$, the generator’s loss function becomes constant and stops learning. But for the other generators, the discriminator is not optimal, and these generators will continue to learn through competing with the discriminator. The updated discriminator will turn into the non-optimal solution, and the generator $G_{k}$ will resume learning. Longer and more stable training periods means better feature extraction capabilities for the discriminator. In this case, the discriminator can be trained to its optimality.

The discriminator in the standard GANs treats all the real targets as one distribution $P_{\mathrm{data}}$. It just aims to explore the gap between the real and fake data distributions, without paying attention to the distinction between different types of the real data. Therefore, as shown in Figure 1, we add a multi-classifier network in the GANs, which shares the feature extraction layers with the discriminator. It can encourage the low layers of the discriminator to explore the differences between different classes of real images in the feature space.

In this way, the training process of GANs is a competition between three sub-networks: a set of generators, a discriminator, and a multi-classifier. The generators expect the mixture distribution to be as close as possible to the real distribution and generate realistic images, whilst the discriminator is responsible for distinguishing whether these input images are real or generated and whose input includes the labeled, unlabeled, and generated images. The multi-classifier is designed to ensure that the input labeled samples are given the right labels. Similar to the standard GANs, D, C, and $G_{1:K}$, in our network, are alternatively updated during the training. The whole loss function of the MCGAN network should be

(6) $$\begin{array}{ll} \underset{G_{1:K},C}{\min}\;\underset{D}{\max}\;V\left(G_{1:K},D,C\right)= & E_{x\in P_{data}\left(x\right)}\left[\log D\left(x\right)\right]+E_{z\in P_{z}\left(z\right)}\left[\log\left(1-D\left(\sum_{i=1}^{K}G_{i}\left(z\right)\right)\right)\right]\\ & -\sum_{j=1}^{N}E_{x\in P_{L}^{j}\left(x\right)}\left[\log C_{j}\left(x\right)\right] \end{array}$$

where $P_{data}\left(x\right)$ refers to the distribution of the real data, including both the labeled and unlabeled samples; $P_{L}^{j}\left(x\right)$ represents the distribution of j_th_ class of the samples; N is the number of the classes; and $C_{j}\left(x\right)$ is the probability that x is class j. The first term of the right hand side of Equation (6) is the same as the first term of Equation (1); and the second term is to ensure that the images produced by all the generators can be correctly distinguished by the discriminator. The last term is a standard Softmax loss for the multi-classifier, which intends to maximize the entropy for the classifier.

**Figure 3:**
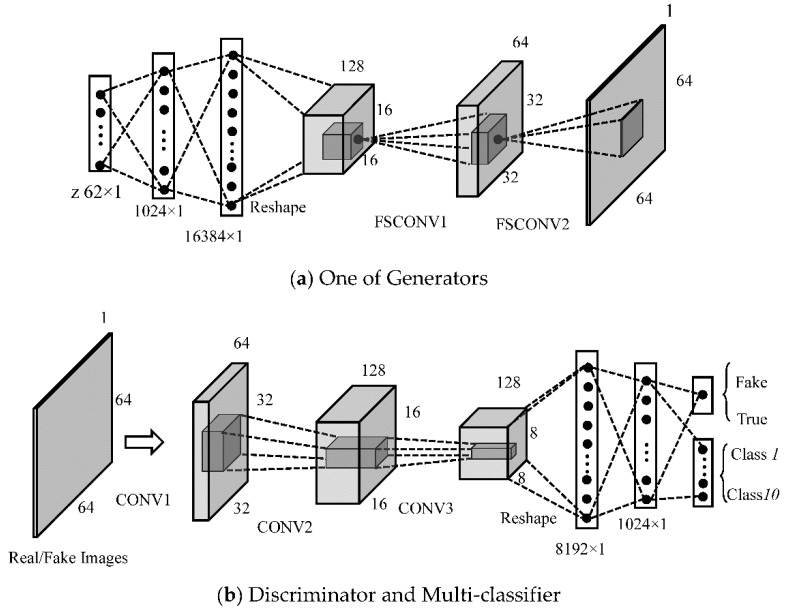
Architecture of the MCGAN for 64 × 64 SAR images: (**a**) One of Generators and (**b**) Discriminator and Multi-classifier.

Various image sizes make the architecture of GANs different. Figure 3a,b show the architecture of our proposed GANs when the input is 64 × 64. The whole GANs includes five generators. The generator mainly consists of two fully connected layers and three convolution layers. The input is a 62 × 1 noise, and its dimension becomes 16,384 × 1 after going through two fully connected hidden layers. Then, the feature vector is reshaped to a 128 × 16 × 16 feature metric, where 128 is the number of the feature maps and 16×16 represents the size of each feature map. Fractionally-strided convolutions are then carried out with 64 convolution kernels (Kernel Size = 4, Stride = 2, Padding = 1) to obtain a set of 64 × 32 × 32 feature vectors, which is followed by a similar fractionally-strided convolution operation to output a 1 × 64 × 64 fake SAR image.

The discriminator consists of three 2Dconvolution operations (Kernel Size = 4, Stride = 2, Padding = 1) and two fully connected hidden layers, where the number of the convolution kernels in two convolution layers are 64 and 128, respectively. All layers take Leaky ReLU as the active function, except for the output layer (Sigmoid).

### 3.2. Architectureof Semi-Supervised Recognition forSAR Images

In this section, we introduce a GANs-based recognition network for identifying 64 × 64 SAR targets. The feature extraction part of the recognition network is derived from the trained low level layer of the discriminator shown in Figure 3b, including three convolution layers and two full connection hidden layers (Dense (8192 × 1)) and Dense (1024 × 1). The classifier includes a 10 × 1 fully connected layer. In the experiments, inspired by the conclusion of Reference [22], we employ the ReLU as the activation function in all the layers of the recognition network.

Finally, we here use a small number of labeled images to train the classifier of the network. The whole procedure of training the MCGAN and the semi-supervised recognition network iteratively is presented in Table 1. In the Results section, we will compare our recognition performance with the standard supervised CNN, DCGAN, and the traditional semi-supervised models, i.e., Label Propagation (LP) [31] and Label Spreading (LS) [32]). LP is a semi-supervised classification algorithm based on graph models. All the labeled and unlabeled images are used as well as the label information that propagates in the graph model until all the images are given appropriate labels. The LS model is similar to the basic label propagation algorithm, but uses an affinity matrix based on the normalized graph Laplacian and soft clamping across the labels. 

## 4. Results

In this section, we first describe the data sets. The comparison and analysis of the proposed model against those of the state-of-the-art semi-supervised and supervised recognition methods are then discussed.

### 4.1. Description of the Data Sets

**Figure 4:**
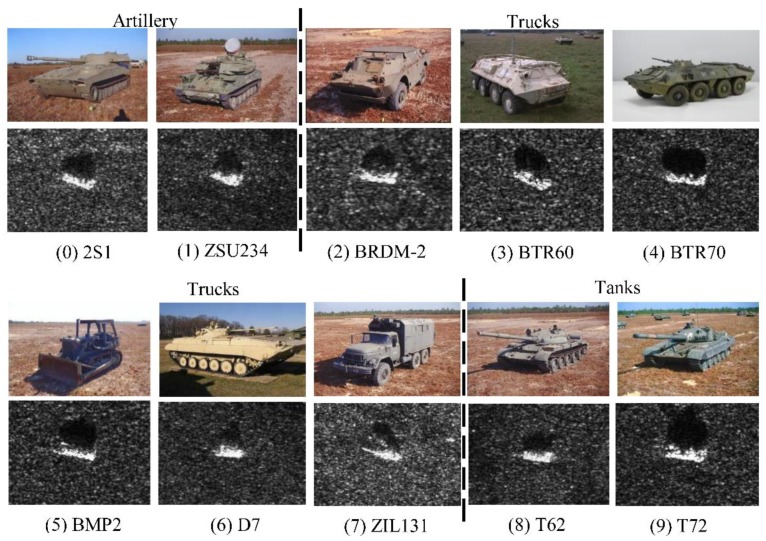
Optical and SAR images of 10 classes of targets in the MSTAR database.

The MSTAR public database was collected using the Sandia National Laboratories Twin Otter SAR sensor payload operating at X band, spotlight mode, and HH single polarization. The MSTAR data contains 10 kinds of vehicle targets. Their SAR and optical images are shown in Figure 4. The SAR images are 128 × 128 and the resolution is 0.3 m. As shown in Figure 4, the radar images of different vehicles are visually indistinguishable. The similar spatial features and the interference from strong speckles and clutters both increase the difficulty of image recognition. 

In the experiment, we train and test our identification network using 128 × 128 and 64 × 64 images. The 64 × 64 images are the pixels in the central part of the128 × 128 images [33]. 10 classes of vehicles can be divided into three major categories, namely, artillery (2S1 and ZSU234), trucks (BRDM-2, BTR60, BTR70, BMP2, D7, and ZIL131), and tanks (T62, T72). Table 2 shows the number of 10 types of the targets in the training and test sets, respectively. 

### 4.2. Experimental Results

In this section, we first present the training procedure of MCGAN using 600 labeled images and 2147 unlabeled samples from the training set. Then, the recognition performance of the MCGAN-based recognition network is compared with LP, LS, standard CNN, DCGAN, DRAGAN, and WGAN.

#### 4.2.1. Training of MCGAN with 600 Labeled Images

**Figure 5:**
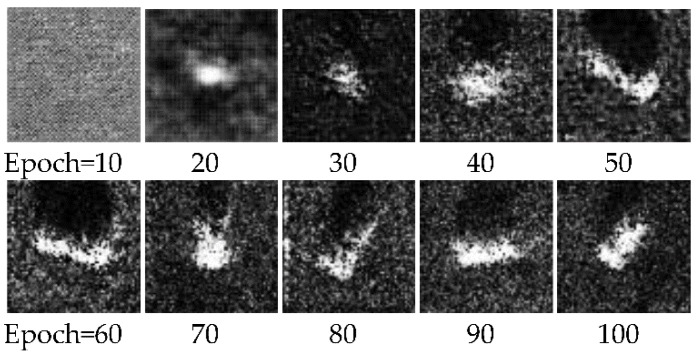
The fake images from one generator with the increase of training time.

In the MCGAN’s training phase, we use all 2747 real SAR images to train our GANs, 600 of which are labeled. All the labeled images are randomly selected from the whole original training set and the numbers of the labeled images of each class are equal. There are five generators. For the 64 × 64 images, the corresponding architectures of GANs and the recognition network have been shown in Figure 3. For the 128 × 128 images, their GANs and recognition networks have the same architecture and hyper-parameters, except for the input layers. Each batch contains 64 images. Generally, we find that GANs can produce highly realistic images after the system runs 100 epochs. Figure 5 shows the faked images from one generator during the training progress. It can be seen that the faked images gradually approach the real images. When the epoch is 100, the visual quality of the generated images has improved.

**Figure 6:**
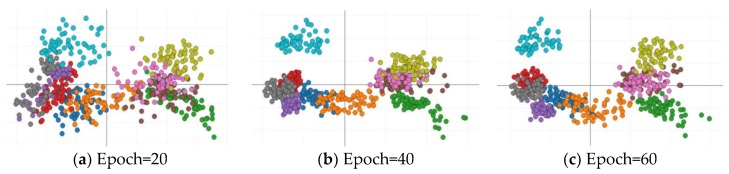
The distribution of feature points from the multi-classifier of MCGAN in different epochs (64 × 64 input): (**a**) Epoch = 20, (**b**) Epoch = 40, and (**c**) Epoch = 60. Different colors represent different categories. With increasing the training epoch, the diverse classes of feature points are gradually separated, indicating the low level layers of the discriminator having learnt the category information from the labeled samples during the training of MCGAN.

In order to evaluate the ability of the low level layers in the multi-classifier to distinguish different kinds of features during the MCGAN training process, we extract the 1 × 10 feature vectors from the output of the multi-classifier and then transform it to two-dimensional using Principal Component Analysis (PCA) in each epoch. The distributions of 10 kinds of feature points for the 64 × 64 input images, when the epoch is 20, 40, and 60, are given in Figure 6, where different colors represent different categories. It can be seen that when the epoch = 20, there are a lot of overlaps between different classes of feature points, whilst various feature points obviously begin to gather around the center of each other when the epoch = 60, and the boundaries between different classes of points become significant.

**Figure 7:**
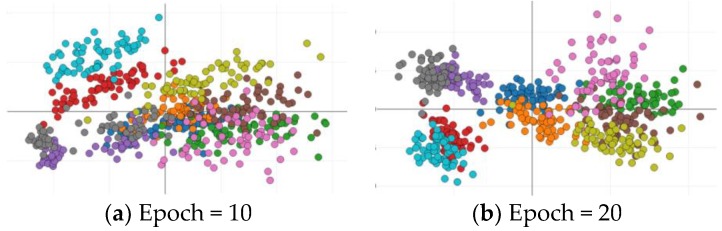
The distribution of feature points from the multi-classifier of GANs in different epochs (128 × 128 input): (**a**) Epoch = 20 and (**b**) Epoch = 40. Different colors represent different categories. When the epoch is 20, the features from the output of low level layers of the discriminator have better separability.

Figure 7 shows the distribution of the feature points under diverse epochs when the input size is 128 × 128. Compared to the case of epoch = 10, different kinds of feature points have better separability when the epoch = 20. This indicates that the addition of the multi-classifier makes the feature extraction layers of discriminator better to distinguish different kinds of samples at some level in the feature space. However, it also should be noted that there are still some feature points from different classes overlapping. This is because these feature extraction layers also need to distinguish between real and fake images, which hinder their learning results. Therefore, for the recognition network that inherits the weights of these feature extraction layers, it needs to further be tuned to improve the recognition accuracy.

#### 4.2.2. Recognition Results Trained with 600 Labeled Images

For both 64 × 64 and 128 × 128 inputs, the semi-supervised identification networks have the same architecture as shown in Figure 3b, except the input layer. The performance of the network is verified by the test dataset. The identification network shares the weights with the three convolution layers and the two fully connected layers of the trained MCGAN. The max training epoch is 100 and the size of the batch is 64. 

**Figure 8:**
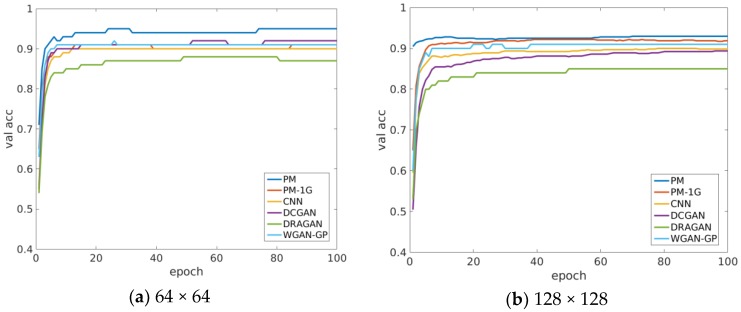
The comparison of the proposed recognition network (PM), supervised CNN, DCGAN, DRAGAN, and WGAN-GP: average validation accuracy for (**a**) 64 × 64 and (**b**) 128 × 128 MSTAR dataset, respectively.

Figure 8 shows the average validation accuracy of the proposed method (PM) in different training epochs. In order to accurately evaluate its classification performance, we run the training 10 times and obtain the mean accuracy values. For comparison, the results of the other five methods are also illustrated in Figure 8, i.e., supervised CNN, DCGAN, WGAN-GP, and DRAGAN. 

The architecture and hyper-parameters of the CNN network are the same as those of our recognition model. The only difference is that its weights are randomly initialized, rather than migrating from the discriminator of MCGAN. For DCGAN, WGAN-GP, and DRAGAN, we first train their corresponding GANs with 600 labeled images. As mentioned in Section 2, DCGAN, WGAN-GP, and DRAGAN all employ the fractionally-strided convolutions and strided convolutions to construct their GANs. The difference is the loss functions of the discriminators and generators. In our experiments, the three GANs models have the same architecture and hyper parameters as the MCGAN show in Figure 3, except that they only have one generator and do not contain a multi-classifier. Then, the output layers of their discriminators are replaced by multi-classifiers (soft-max), which constitute the corresponding recognition networks. The optimizers used in all the networks (G, D, and recognition nets) training are Adam. Specifically, the learning rates of all generators, discriminators, and identification networks are 0.0002.

In Figure 8, the validation accuracy of the proposed model reaches the maximum faster than the other four models for two sizes of the input images, indicating that the introduction of the multi-classifier in MCGAN effectively speeds up the training process of the recognition network. After the epoch = 20, the accuracy is also higher than others. The curve of PM-1G shown in Figure 8 refers to the mean validation accuracy of the identification network—if only one generator is used in MCGAN. It can be seen that PM-1G has a lower recognition accuracy than ours when the epoch is >10, which indicates that the introduction of the multiple generators does improve the feature extraction ability of the discriminator.

Table 3 shows the average accuracy and the corresponding standard deviation (STD) of each method after training the system 10 times. As can be seen, our method’s average accuracy is 92.89% when the input size is 64 × 64, higher than that of the other models. The STD accuracy of the PM and PM-1G are both smaller than in others, indicating the positive role of the multiple generators in the MCGAN’s training. For 128 × 128 inputs, PM similarly has than highest mean accuracy and the lowest STD than other models.

**Figure 9:**
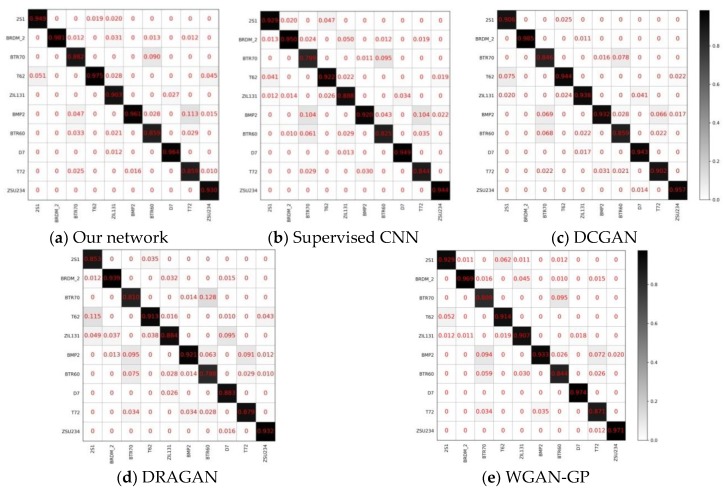
The average confusion matrix of (**a**) our network; (**b**) supervised CNN; (**c**) DCGAN; (**d**) DRAGAN; and (**e**) WGAN-GP (64 × 64). The average classification accuracy of our method is higher than the other four methods. Moreover, for some targets that are difficult to identify, such as BTR60 and BTR70, due to the efficient learning in the training of GANs, the classification accuracy of ours, for BTR60 and BTR70, are both more than 86%, whilst that of the other four methods are below 85%.

Figure 9 shows the average confusion matrix of the above five methods for 64 × 64 inputs after the system has been trained 10 times. Each element of the matrix is the mean of the corresponding elements of 10 confusion matrices. It can be seen that the minimum classification accuracy of our method is 85.9% for BTR70, while those of CNN, DCGAN, DRAGAN, and WGAN-GP are 82.5%, 85.9%, 78.8%, and 84.4%, respectively. Experimental results show that the stable training of MCGAN effectively improves the features extraction ability of the discriminator. Hence, the recognition network is able to achieve satisfactory accuracy even in the case of poor training.

Cohen’s kappa coefficient (κ) [34] was also introduced to measure the correctness of the classification of the above multiple recognition networks in Table 4. The definition of a Kappa coefficient is

(7) $$\frac{p_{o}-p_{e}}{1-p_{e}}$$

where $p_{o}$ is the relative observed agreement between the classifier’s results for the test data and the real labels; $p_{e}$ is named as the hypothetical probability of the chance agreement. The closer that this coefficient is to one, the better the agreement between the classification results and the ground truth is. As shown in Table 4, our recognition network for two sizes of inputs has both the highest Kappa coefficients than the other five methods, which are 0.922 and 0.919. These results are consistent with the mean accuracy of the six methods shown in Table 3.

#### 4.2.3. Recognition Results Trained by Different Numbers of Labeled Images

**Figure 10:**
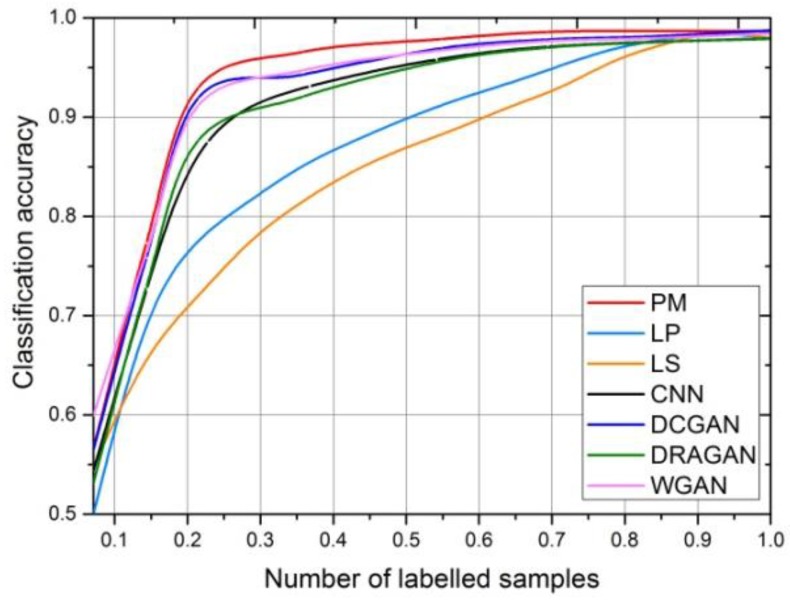
The average recognition accuracy of MCGAN, supervised CNN, DCGAN, DRAGAN, LS, LP, and WGAN-GP trained using different numbers of labeled images (64 × 64).

In order to further evaluate the performance of the proposed method, the mean recognition accuracy of the proposed method, trained using different numbers of the 64 × 64 labeled images, is illustrated in Figure 10. The proportion of the labeled samples used in training GANs and fine-tuning the recognition networks, in all the real samples, gradually increases from 0.073 to one. Similarly, we ran the training 10 times for each method and calculated the average accuracy. In Figure 10, the horizontal represents the proportion of the labeled images in the training set, and the vertical is the average classification accuracy. As a comparison, we also produced the results of LP [31], LS [32], DCGAN, DRAGAN, and WGAN-GP. As the LP and LS algorithms cannot directly process two-dimensional images, PCA was first introduced to code the images into 1 × 1024 feature vectors. 

Due to the powerful feature extraction ability of the discriminator, our method has a higher classification accuracy than DCGAN, DRAGAN, and WGAN-GP, especially when the proportion of the labeled sample is between 20% and 90%. In particular, our accuracy can be more than 92% in the case of the 20% labeled images. The LP and LS methods were not performing well in the case of the fewer labeled images because the speckles and clutters in the SAR images affected the construction of their graph models. When the proportion was more than 90%, the classification accuracy of the seven methods was close (over 98%), which meant that about 50 or less test images were difficult to correctly classify.

**Figure 11:**
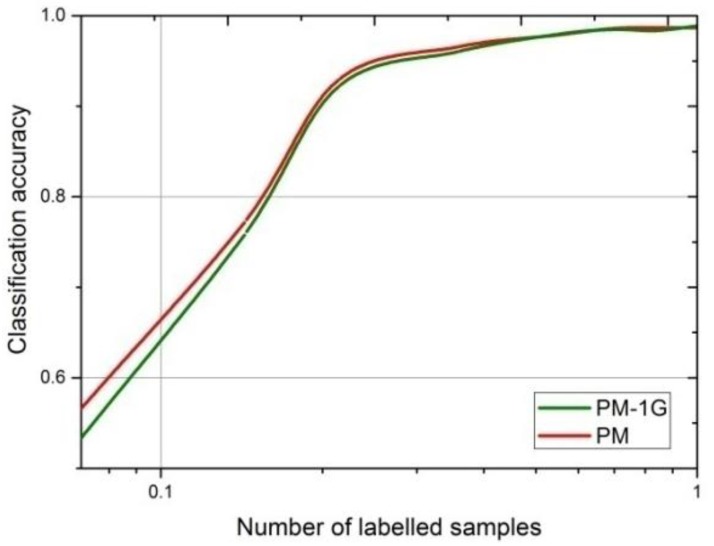
The average recognition accuracy of our network and PM-1G trained using different numbers of labeled images (64 × 64).

Figure 11 compares the recognition accuracy of our method and the PM-1G trained using different numbers of 64 × 64 labeled images, where the proportion of the labeled images increased exponentially. It can be seen that the reduction of the generator in PM-1G affects the training of the discriminator in the GANs, resulting in a decrease in the recognition performance of the identification network, especially when the proportion of the labeled samples is less than 20%.

#### 4.2.4. Choosing the Number of the Generators of MCGAN

How many generators used in the MCGAN is appropriate? In this part, we compare the average training time, the mean classification accuracy, and the Std of MCGAN in the case of using different numbers of generators in Table 5. Note that the training time here refers to the mean training time of an epoch. The main configuration of our computer is 62G Memory and Intel Xeon(R) CPU E5 @ 3.5GHz×8 (Santa Clara, CA, USA). The graphics are GeForce GTX TITAN X/PCle/SSE2 (Santa Clara, CA, USA). The structure and hyper-parameters of MCGAN are the same as the description presented in Section 4.2.2. Training is performed using 400 labeled 64 × 64 images, and the classification accuracy and Std are obtained after training and testing 10 times. It can be seen that, as the number of the generators increases from one to five, the average classification accuracy increases from 75.76% to 77.13%; the Std gradually decreases. However, the accuracy does not continue to increase when the generator increases to eight. In addition, we found that each additional generator increases the training time in an epoch by three-to-four seconds. Redundant generators seriously affected the efficiency of the models. Therefore, we employed five generators in the MCGAN in this paper.

#### 4.2.5. Running Time

To evaluate the computational efficiency of our method, we compare the average computational time of each step of our method with the other five methods in Table 6. The whole training procedure can be divided into the training of GANs and the training of the recognition networks. Hence, we first compare the mean computational time of training the GANs of six models in an epoch in Table 6. The computational time of MCGAN is triple that of the other five models due to the increased number of generators. The third column of Table 6 gives the training time of the corresponding six GANs-based recognition networks. These networks have the same hyper-parameters and structure, except the initial weights, so their training time should be the same, in theory. The results of the experiment also confirm this view, and their training time in an epoch is approximately 0.26s. 

## 5. Discussion

For the application of GANs-based semi-supervised recognition methods in the SAR images, two issues need to be considered: (1) How to enable the GANs to learn the class information from the labeled samples; and (2) how to avoid the training of the GANs being influenced by the strong speckles in the SAR images.

For the first problem, the dominant semi-supervised learning method based on GANs is proposed in Reference [19], which changes the output layer of the trained discriminator from a binary classifier to a multi-classifier, and then employs the labeled samples to fine-tune the new network. Its disadvantage is that the GANs cannot learn the label information of the input images during the adversarial training process, which limits the further improvement of the recognition accuracy. In this paper, we add a multi-classifier in the discriminator, so that the low level layers of the discriminator can learn the category information recurrently in the competition between the discriminator and the generator.

For the second problem, we introduced a number of independent generators in the GANs. The instable training of the standard GANs is because there is no non-negligible intersection between the distributions of the discriminator and generator in the feature space when the discriminator is almost optimal, causing the generator to stop updating. In the case of multiple generators, even if the distribution from the discriminator does not intersect with one of the generators, the distributions of the other generators still keep a non-negligible intersection with the discriminator’s distribution, ensuring that the GANs continue to update. 

This method plays a similar role to the method reported in Reference [29] in the training of the GANs, which fixes the collapsing issues by adding continuous noise to the inputs of the generator. It has been proven that the noise will increase the probability that the discriminator’s distribution $P_{data+\gamma }$ and the generator’s distribution $P_{g+\gamma }$ have overlaps, where γ represents an absolutely continuous random variable [29]. Multiple generators can also increase the chance that the generator’s and the discriminator’s distributions have intersections. In this case, the discriminator can be trained until the optimality has been reached.

The experimental results prove that the above two aspects effectively improve the recognition performance—compared with the standard GANs-based semi-supervised method shown in Reference [19]. Firstly, in order to prove the role of the multiple classifiers in GANs, we compared our model with one generator (PM-1G) with the standard DCGAN-based semi-supervised recognition method [19]. Experiments show that even though only one generator is employed, the multi-classifier can apparently improve our classification accuracy. Secondly, experimental results also demonstrate that the multiple generators further increase the average classification accuracy for 10 categories of SAR targets.

## 6. Conclusions

This paper has presented a novel semi-supervised GANs model for SAR image recognition. First, the multiple generators are introduced into the GANs to enhance the feature extraction capability of the discriminator. Secondly, a multi-classifier is employed to ensure that the discriminator can explore the gaps between different classes of images in the feature space while distinguishing whether or not the input is an authentic image. We provide the evidence that our MCGAN can learn good representations of SAR images from the unlabeled and labeled images simultaneously. In the application of recognition, low level layers of the trained discriminator are utilized as a feature extractor of a recognition network, which is further tuned using the same set of the labeled images. Experiments on the MSATR data have proved that our model achieved better classification accuracy and maintained a stable recognition performance, in comparison to the supervised CNN, DCGAN, DRAGAN, WGAN-GP, and the other traditional semi-supervised models. 

## Figures and Tables

**Table 1:** Training the semi-supervised recognition network for SAR images.

Step1: Training the GANs for SAR images	
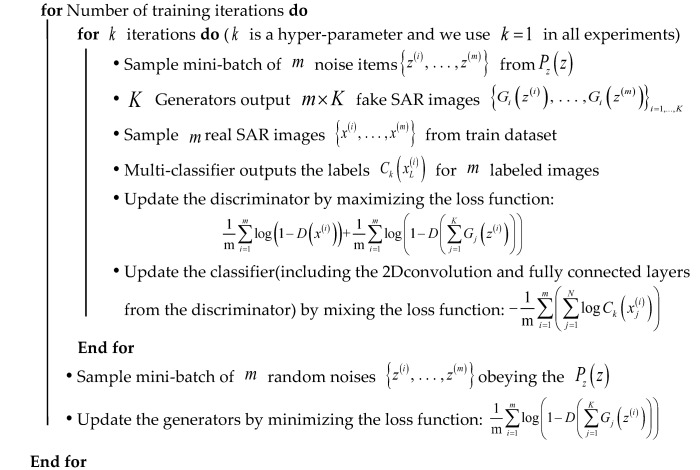	
Step2: Training the Recognition Network for SAR images	
**for** Number of training iterations **do**	
	Initialize the recognition network using the weights of discriminator Sample M labeled SAR images $\left\{x^{\left(i\right)},\ldots ,x^{\left(M\right)}\right\}$ from train datasets Update the recognition network by descending its stochastic gradient
**End for**	

**Table 2:** The number of images from 10 kinds of targets in the training and test sets.

Type	Train Data	Test Data
2S1	299	274
ZSU234	299	274
BRDM-2	298	274
BTR60	256	195
BTR70	233	196
BMP2	233	195
D7	299	274
ZIL131	299	274
T62	299	273
T72	232	196
Total	2747	2425

**Table 3:** The mean accuracy and STD of our network, supervised CNN, DCGAN, DRAGAN, and WGAN-GP trained by 600 labeled images.

Method	64 × 64		128 × 128	
	AC	Std	AC	Std
PM	**92.89%**	0.473%	**90.19%**	**0.355%**
PM-1G	92.15%	**0.379%**	89.10%	0.733%
DCGAN	91.87%	0.639%	87.16%	0.594%
CNN	89.58%	0.527%	89.16%	0.771%
DRAGAN	87.58%	0.504%	84.25%	0.407%
WGAN-GP	91.11%	0.557%	89.59%	0.666%

**Table 4:** The Cohen’s kappa coefficient (κ) of PM, PM-1G, DCGAN, supervised CNN, DRAGAN, and WGAN-GP trained by 600 labeled images.

**Method**	**Kappa Coefficient (κ)**	
	**64 × 64**	**128 × 128**
PM	**0.922**	**0.919**
PM-1G	0.920	0.899
DCGAN	0.916	0.877
CNN	0.888	0.897
DRAGAN	0.869	0.845
WGAN-GP	0.907	0.906

**Table 5:** The mean accuracy, STD, and training time of MCGAN with a different number of generators when trained by 400 labeled images.

Number of Generators	AC	Std	Training Time (s/epoch)
1	75.76%	0.66%	5.1
2	75.63%	0.47%	8.3
3	76.08%	0.62%	12.1
4	75.93%	0.50%	15.6
5	**77.13%**	0.55%	17.9
6	76.89%	0.28%	21.4
7	77.00%	0.42%	24.3
8	76.52%	0.74%	27.1

**Table 6:** The training time of our network, supervised CNN, DCGAN, DRAGAN, and WGAN-GP trained by 600 labeled images.

Method	Training GANs(s/epoch)	Training Recognition Nets(s/epoch)
PM	17.9	0.28
PM-1G	5.1	0.26
DCGAN	5.1	0.26
CNN	-	0.26
DRAGAN	7.7	0.28
WGAN-GP	5.51	0.26
